# Psychopathology Related to Energy Drinks: A Psychosis Case Report

**DOI:** 10.1155/2017/5094608

**Published:** 2017-01-02

**Authors:** Daniel Hernandez-Huerta, Maria Martin-Larregola, Jorge Gomez-Arnau, Javier Correas-Lauffer, Helen Dolengevich-Segal

**Affiliations:** ^1^Department of Psychiatry, Ramon y Cajal University Hospital, Madrid, Spain; ^2^Department of Psychiatry, Henares University Hospital, Coslada, Madrid, Spain; ^3^Francisco de Vitoria University, Madrid, Spain

## Abstract

Energy drinks (ED) are nonalcoholic beverages that have caffeine as their most common active substance. The rapid expansion of ED consumption has created concern in the scientific community as well as in the public opinion. We report a psychotic episode probably triggered by ED abuse in a young adult without previous psychotic disorders. We have reviewed the literature regarding the relationship between caffeine, energy drinks, and psychopathology. Few articles have been published about mental health effects of energy drinks and caffeine abuse. Nevertheless, this relationship has been suggested, specifically with anxiety disorders, manic episodes, suicide attempts, psychotic decompensation, and substance use disorder. ED consumption could represent a global public health problem because of the potential severe adverse effects in mental and physical health. To our knowledge, this article is probably the first case of psychosis related to ED abuse in an individual without previous psychotic disorders.

## 1. Introduction

ED constitute a relatively new product category in the wide soft drink market. The category includes a variety of nonalcoholic beverages marketed for their perceived effects as stimulants, energizers, and performance enhancers. The most common substance in ED is caffeine, which is often combined with taurine, D-glucurono-y-lactone, guaraná, maltodextrin, ginseng, carnitine, creatine, and gingko biloba; other common ingredients are vitamins and artificial and natural sweeteners [1]. Hundreds of different brands are now marketed with significant variation in their caffeine content (ranging from a modest 50 mg to an alarming 505 mg per can or bottle) and caffeine concentration (ranging from 2.5 mg to 17.1 mg per fluid ounce). The acute and long-term effects resulting from excessive and chronic consumption of ED are not yet fully known [2, 3].

The rapid expansion of ED consumption has created concern in the scientific community as well as in the public opinion. Available information suggests that ED consumption is becoming more and more widespread among young individuals, especially in relation to entertainment and sports practice. In the European Union, the highest prevalence of consumption was observed in the adolescent age group, with 68% having consumed at least once in 2012. However, prevalence of consumption in adults was 30% and in children was 18% [4, 5].

Caffeine seems to be the main ED compound to produce a stimulant effect [6, 7]. Caffeine can influence the activity of neuronal control pathways in the peripheral and central nervous system (CNS). The neuropsychiatric effects of caffeine are mediated by antagonism of adenosine A_1_ and A_2A_ receptors in the CNS. As adenosine inhibits dopaminergic neurotransmission, blockage of A_2A_ receptors by caffeine may increase dopaminergic activity. Antagonism of A_1_ receptors regulates the release of neurotransmitters such as glutamate or acetylcholine. Caffeine differs from classical substances of misuse in causing dopamine release in the prefrontal cortex rather than the nucleus accumbens [8].

## 2. Clinical Case

The patient was an 18-year-old Spanish male with no relevant medical or psychiatric history. Regarding his substance use, he admitted daily use of tobacco (20 cigarettes/day, meeting criteria for tobacco use disorder), daily use of cannabis (3 cannabis cigarettes/day, meeting criteria for cannabis use disorder), and occasionally weekend drinking (no meeting criteria for alcohol use disorder). He denied consumption of other psychoactive substances. As for his family background there was a paternal uncle with an unspecified chronic mental illness.

He was admitted to a psychiatry ward after presenting with an acute psychotic episode. The clinical picture included delusions of reference and persecution, pressured speech, increased alertness, fright, suspiciousness, marked anxiety, and psychomotor agitation during previous days. Hallucinations were not identified. He was aware and oriented in person, place, and time. Over the previous week, he had felt very nervous due to the proximity of high school exams. Thus, he had been drinking about 6 ED cans (80 mg caffeine per can) per day during the last seven days. Also, he had been sleeping less than three hours per night in this period, until finally he showed global insomnia during the day prior to hospital admission. He denied increasing cannabis smoking in these days or consumption of other substances.

During his first day in the inpatient unit he presented severe acute psychomotor agitation that required mechanical restraint and high doses of medication (first olanzapine 20 mg oral, followed by haloperidol 5 mg and levomepromazine 25 mg intramuscularly). After these treatments he exhibited low awareness and the ECG showed sinus bradycardia and QTc prolongation. He stayed 24 hours in the intensive care unit until his ECG was normalized and he recovered good awareness level. Urinary drug screening test was positive for cannabinoids and negative for opioids, benzodiazepines, cocaine, amphetamines, tricyclic antidepressants, and barbiturates. Additional tests (cranial CT and blood tests) were normal.

The patient returned to the psychiatry ward and was treated with olanzapine 10 mg daily and there was cessation of caffeinated drinks. After three days, psychotic symptomatology had disappeared and he was discharged with diagnosis of Substance-Induced Psychotic Disorder (according to DSM-5 classification) relative to stimulants (ED) and cannabis. He was followed up at a mental health outpatient setting with positive global evolution. He remained abstinent to cannabis and ED. Antipsychotic treatment was gradually reduced and he was finally discharged of mental health service without pharmacological treatment two years later.

## 3. Discussion

Five caffeine-related syndromes are recognized in the* Diagnostic and Statistical Manual of Mental Disorders, Fifth Edition* (DSM-5) [9]; however, the International Classification of Diseases Tenth Revision (ICD-10) is less specific and only recognizes “mental and behavioural disorders due to use of other stimulants, including caffeine” [8] (Table 1).

Caffeine intake has been linked as a potential risk factor for a wide range of psychiatric and substance use disorders. A genetic factor seems to predispose people to both caffeine intake and the risk for psychiatric disorders [10]. For example, it has been described that individuals with the 1976T/T genotypes for A2A adenosine receptors showed higher levels of anxiety after caffeine administration than other genotypic groups [11, 12].

Some studies have demonstrated that the intake of large amounts of caffeine is associated with psychotic and manic symptoms and/or exacerbation of previous psychotic symptoms [13]. Case reports have also suggested that excessive caffeine intake may hamper the recovery of patients with bipolar disorder or manic-type mood episodes. For this reason, several guidelines recommend discontinuation of caffeine intake as one of the first steps in the treatment of mania [7].

Case report evidence suggests that caffeine might induce psychotic symptoms also in some individuals without previous psychotic disorders, but this has not yet been confirmed. This may result from an exacerbation of underlying paranoid traits. Also, reduction of caffeine intake has been associated with symptom improvement in patients with psychotic disorders [7]. Individuals with a predisposition to psychosis may have a greater disposition or demonstrate a lower threshold to develop psychopathology following ingestion of caffeine [14].

Recently, case reports of psychopathology related to ED consumption have been reported (Table 2).

Initial evidence to suggest that ED use can be associated with the occurrence/reoccurrence of psychiatric symptoms comes from these case reports. Although these cases imply that excessive consumption of energy drinks may act as a trigger for relapse in certain vulnerable people with preexisting mental health problems, cases have also emerged in which serious psychiatric symptoms have occurred in otherwise healthy individuals [15].

In a recent study, Marmorstein [16] examined the association between ED and coffee consumption and psychopathology among early teenagers. The author studied symptoms of depression, anxiety, conduct disorder (CD), and attention deficit hyperactivity disorder (ADHD) and found that ED and coffee consumption were concurrently associated with similar psychopathology symptoms. However, longitudinally, the associations between these beverages and psychopathology differed. Specifically, ED consumption at the initial assessment predicted increases in CD and ADHD rates. Conversely, initial levels of ADHD hyperactivity symptoms predicted later increases in coffee consumption, while social anxiety was protective against increases in energy drink consumption [16].

Richards and Smith [15] have recently reviewed the literature about the chronic effects of ED on mental health. They concluded that although acute mood effects associated with ED appear often to be positive, chronic use also tends to be associated with undesirable mental health effects such as stress, anxiety, and depression. However, as almost all identified studies were cross-sectional and some did not control for other relevant factors such as sex, socioeconomic status, and additional caffeine intake, the nature of this relationship is not yet fully understood [15].

In this case report we hypothesize that psychotic symptoms could be related to the excessive consumption of ED during the days prior to hospitalization. Review of the topic shows similar case reports although there is not enough literature yet about the psychiatric effects of ED. Otherwise, we have to take into account other important factors that could play an important role in this case. These relevant factors are cannabis use disorder and sleep deprivation. Both have been linked with psychosis in several researches and, therefore, they have been able to contribute as etiologic and pathogenic factors.

Current cannabis abuse or dependence increases the risk of transition into psychosis in persons at ultrahigh risk of psychosis [17, 18]. There have been few studies addressing the effects of caffeine and cannabis in combination, but they have been focused on their combined effects on memory but not in psychosis [19]. Our patient met criteria for cannabis use disorder and is important to take it into account in this psychotic process. However, psychotic symptoms only appeared after the excessive consumption of ED and, thus, cannabis could not be considered as unique etiologic factor.

Excessive intake of caffeinated ED leads to sleep deprivation [20], which also has implications for psychotic pathogenesis. Sleep deprivation has been associated with an increase in self-reported psychiatric symptoms, including somatic complaints, anxiety, depression, mania, and paranoia [21]. Insomnia symptoms have also been related to higher levels of persecutory thoughts, even in the general population [22]. Recently, another study has shown that sleep deprivation induces sensorimotor gating deficits and elevated self-reported psychosis-like experiences in healthy humans [23]. In our case report, sleep deprivation is probably consequence of excessive consumption of ED and excessive tension due to exam stress. This effect, in accordance with the other relevant factors, has been able to influence the emergence of psychotic symptoms.

It is also remarkable that the patient suffered a severe cardiovascular complication (sinus bradycardia and QTc prolongation) after antipsychotic drug administration. A possible QTc prolongation secondary to antipsychotic drugs is well-known [24, 25]. However, cardiovascular effects, such as supraventricular tachycardia, long QT syndrome, or myocardial infarction, have also been described after ED intake [26–29]. In addition, a number of investigations have focused on the hemodynamic effects and endothelial functions of the consumption of energy drinks [30–33]. For this reason we hypothesize that the complication described in our patient could be multifactorial and not only related to antipsychotic drugs.

## 4. Conclusions

To our knowledge this case report is probably the first case of psychosis related to ED abuse in a patient without a previous diagnosis of psychotic disorder. ED abuse likely played the most important role in the pathogenesis of psychosis in this case report; however, other factors, such as cannabis use disorder, genetic factors, and sleep deprivation, were probably relevant and facilitated the process.

ED consumption could represent a global public health problem because of its potential severe adverse events. Few articles have been published on this important matter. The general public and potentially vulnerable users, such as adolescents, should be advised that caution is warranted when using these drinks, especially in large quantities over short periods of time or mixed with cannabis, alcohol, or other substances. More research is needed to determine the potential health and mental effects associated with ED consumption.

## Figures and Tables

**Table 1:** Caffeine-related syndromes in DSM-5 and ICD-10.

DSM-5	ICD-10
(1) Caffeine-induced sleep disorder (F15.182, F15.282, F15.982)	(1) Mental and behavioural disorders due to use of other stimulants, including caffeine (F15)
(2) Caffeine-induced anxiety disorder (F15.180, F15.280, F15.980)	
(3) Caffeine intoxication (F15.929)	
(4) Caffeine withdrawal (F15.93)	
(5) Unspecified caffeine-related disorder (F15.99)	

**Table 2:** Case reports published about psychopathology related to ED consumption.

Reference	Previous mental illness	ED consumption	Psychopathology related to ED consumption	Treatment
Cruzado et al. [34]	No	4 cans of Magnus® Omnilife	Manic episode	Risperidone 3 mg/day and ED cessation
		Products + 5 coffee cups, daily, during several weeks		

Szpak and Allen [35]	No	7 cans of unspecified ED during two days	Suicide attempt	ED cessation

Rizkallah et al. [36]	Case 1: bipolar disorder type I and cocaine dependence	6 cans of unspecified ED, daily, during one week	Manic episode	ED cessation
	Case 2: bipolar disorder type II and cocaine dependence	8 cans of unspecified ED, daily, during one month	Manic episode	ED cessation
	Case 3: bipolar disorder type I, cannabis dependence, and cocaine abuse	9 cans of unspecified ED, daily, during two weeks	Mood decompensation	ED cessation

Cerimele et al. [37]	Schizophrenia	10 cans of unspecified ED, daily, during two months	Psychotic decompensation	ED cessation

Berigan [38]	No	6–8 cans of unspecified ED, daily, during four months	Anxiety disorder	ED cessation

Chelben et al. [39]	Case 1: cluster B personality disorder	5 cans of unspecified ED, daily, during 1 week	Mood decompensation	ED cessation
	Case 2: bipolar disorder, borderline personality disorder, and multiple substance abuse	5–10 cans of unspecified ED, daily, during one month	Mood decompensation	ED cessation
	Case 3: schizophrenia	8-9 cans of unspecified ED, daily, during one month	Mood decompensation	ED cessation

Machado-Vieira et al. [40]	Bipolar disorder type I	6 cans of Red Bull® in total during 1 week	Manic episode	ED cessation

Menkes [41]	Schizophrenia	5 cans of Demon Shot® in total during 1 week	Psychotic decompensation	ED cessation

Sharma [42]	No	6–8 cans of Red Bull (550 mL) during 1 week	Manic episode	Olanzapine 10 mg/day and ED cessation
